# Joint Modeling of Singleton Preterm Birth and Perinatal Death Using Birth Registry Cohort Data in Northern Tanzania

**DOI:** 10.3389/fped.2021.749707

**Published:** 2021-11-30

**Authors:** Innocent B. Mboya, Michael J. Mahande, Joseph Obure, Henry G. Mwambi

**Affiliations:** ^1^School of Mathematics, Statistics, and Computer Science, University of KwaZulu-Natal, Pietermaritzburg, South Africa; ^2^Department of Epidemiology and Biostatistics, Institute of Public Health, Kilimanjaro Christian Medical University College, Moshi, Tanzania; ^3^Department of Obstetrics and Gynecology, Kilimanjaro Christian Medical Center, Moshi, Tanzania

**Keywords:** preterm birth, perinatal death, joint modeling, bivariate binary outcomes, adverse perinatal outcomes, sub-Saharan Africa

## Abstract

Understanding independent and joint predictors of adverse pregnancy outcomes is essential to inform interventions toward achieving sustainable development goals. We aimed to determine the joint predictors of preterm birth and perinatal death among singleton births in northern Tanzania based on cohort data from the Kilimanjaro Christian Medical Center (KCMC) zonal referral hospital birth registry between 2000 and 2017. We determined the joint predictors of preterm birth and perinatal death using the random-effects models to account for the correlation between these outcomes. The joint predictors of higher preterm birth and perinatal death risk were inadequate (<4) antenatal care (ANC) visits, referred for delivery, experiencing pre-eclampsia/eclampsia, postpartum hemorrhage, low birth weight, abruption placenta, and breech presentation. Younger maternal age (15–24 years), premature rupture of membranes, placenta previa, and male children had higher odds of preterm birth but a lessened likelihood of perinatal death. These findings suggest ANC is a critical entry point for delivering the recommended interventions to pregnant women, especially those at high risk of experiencing adverse pregnancy outcomes. Improved management of complications during pregnancy and childbirth and the postnatal period may eventually lead to a substantial reduction of adverse perinatal outcomes and improving maternal and child health.

## 1. Introduction

Globally, there is a notable decline of under five mortality rates since the year 1990 (1). Despite this decline, the share of mortality burden increased in the group of children in younger ages, especially in the first 28 days of life (neonatal period) (1–3). The United Nations (UN) Inter-agency Group for Child Mortality Estimation report indicated that at a global rate of 17 deaths per 1,000 live births, and approximately 6,700 neonatal deaths everyday in 2019, neonatal period is the most vulnerable time for children under 5 years of age (1). The share of neonatal mortality to under five deaths has increased from 40% in 1990 to 47% in 2019 (1). In addition, sub-Saharan Africa (SSA) caries the highest burden of neonatal mortality rates in the world (1, 2). Most of the neonatal deaths occur during the perinatal period (1, 4, 5). A recent meta-analysis in 21 SSA countries estimated a perinatal mortality rate of 34.7 per 1,000 liver births. The Eastern Africa region had a rate of 34.5 per 1,000 live births, and was highest (39.5 per 1,000 live births) in Tanzania (6, 7).

Preterm birth complications are among the leading causes of perinatal and neonatal deaths (1). In 2018 alone, preterm birth complications accounted for 35% of all neonatal deaths, followed by intrapartum-related complications (24%) (8). Globally, preterm birth rate was 10.6%, equivalent to nearly 15 million live preterm births in 2014, 81% occurring in Asia and SSA (9). If these estimates are left unchecked within and between countries, there may be a proportional increase in perinatal deaths. Currently, Tanzania ranks the tenth country with the highest preterm birth rate in the world (16.6%) and shares a 2.2% of the global preterm birth proportions (9). Timely, quality, and skilled newborn care at birth and treatment immediately after birth and first days of life is essential to increase child survival (1, 9).

Previous studies assessed the independent predictors of preterm birth and perinatal deaths or as the determinants of each other (10–17). Maternal characteristics and conditions and complications in the current pregnancy increase preterm birth and perinatal death risk (18–22). Also, previous exposure to these outcomes increases the recurrence risk (11, 12, 20, 23, 24). These demonstrate the association between preterm birth and perinatal deaths. In other words, the two outcomes within the same individual are highly correlated. Birth registries are examples of such data where several outcomes are highly correlated. Joint modeling is relevant to reveal more about their relationship, hence inform clinical and public health decisions.

Joint modeling, particularly using the random effects approach, have been previously applied to clinical outcomes such as HIV and HCV (25, 26), hearing thresholds (27, 28), and body mass index with other clinical targets among diabetic patients (29). The application of these methods to pregnancy-related adverse outcomes is limited. This study aimed to jointly model preterm birth and perinatal death using the KCMC zonal referral hospital medical birth registry data in northern Tanzania. To our knowledge, no studies have jointly modeled preterm birth and perinatal death in Tanzania. A joint model of the two outcomes will help better understand potential risk factors for early diagnosis and management of high-risk pregnancies.

## 2. Materials and Methods

### 2.1. Description of the Data Source

Data used in this study comes from a prospective hospital-based maternally linked cohort data from the KCMC zonal referral hospital in Moshi Municipality, Northern Tanzania. Details about this birth registry are published elsewhere (14, 15, 17, 30–32). Briefly, the KCMC medical birth records information for women and their subsequent deliveries from 2000 to date. The hospital has an average of 3,500–4,000 births every year, close to 70,000 recorded deliveries to date. All consenting mothers are interviewed using a specially designed questionnaire by the project midwives 24 h after normal delivery. Mothers undergoing cesarean delivery or who experienced a complicated birth are interviewed on the second or third day, depending on their condition. The questionnaire captures information on maternal and paternal background characteristics, mothers' health before and during present pregnancy, delivery-related information and complications, and child status (i.e., whether child is dead or alive). Also, additional data were abstracted from the antenatal care (ANC) cards and the hospital medical records of the mother. Unique identification numbers are used to link mother and child information.

### 2.2. Study Population and Eligibility Criteria

**Figure 1:**
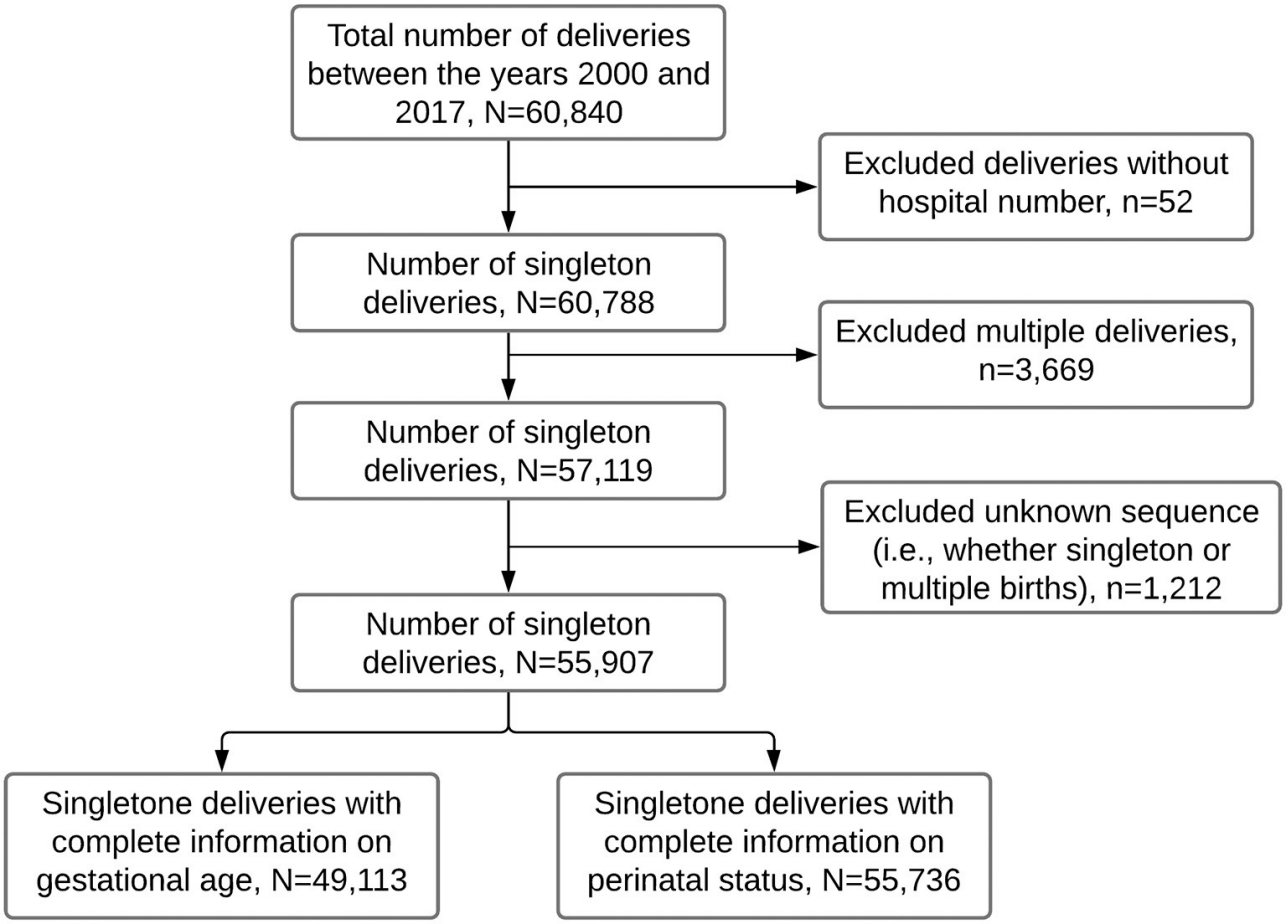
Flow chart showing the number of singleton deliveries analyzed in this study. Data from the Kilimanjaro Christian Medical Center (KCMC) Medical birth registry, 2000–2017.

The study population for this study was women who delivered singleton babies from January 2000 to December 2017. For this period, there were 60,840 deliveries from 45,324 mothers aged 15–49 years. We excluded 52 records missing unique identification numbers (used to link mothers and their subsequent births) and 3,669 multiple gestations (i.e., twins and triplets) to avoid over-representing high-risk pregnancies. We further excluded 1,212 deliveries of unknown sequence (i.e., whether singleton or multiple births). We, therefore, analyzed data for 55,907 recorded deliveries, of which 49,113 had complete information on gestational age and 55,736 on perinatal status (Figure 1).

### 2.3. Study Variables and Variable Definitions

The primary outcomes were preterm birth and perinatal death. Perinatal death comprises stillbirths (pregnancy loss that occurs after 7 months of gestation) and early neonatal death (death of live births within the first 7 days of life) (7, 33). We coded perinatal death as binary, that is, “Yes” if the child died and “No” if otherwise. Preterm birth is any birth before 37 completed weeks of gestation or fewer than 259 days from the first date of a woman's last menstrual period (9, 12, 34) and was also analyzed as a binary variable (<37 vs. ≥37 weeks of gestation).

The secondary outcome was the co-occurrence of preterm birth and perinatal death. We generated a categorical variable from the two outcomes with the following categories; “0” if none of the events occurred, “1” if both occurred, “2” if perinatal death only, and “3” if preterm birth only occurred. We then used a multinomial random-effects regression model to predict the independent and co-occurrence of preterm birth and perinatal death.

The independent variables included maternal and paternal background characteristics and maternal conditions and complications during pregnancy and delivery. Previous literature (18–23) and analyses of this cohort data informed selection of these variables (14, 15). The background characteristics were maternal age (15–19, 20–24, 25–34, 35–39, and 40+), paternal age (15–24, 25–29, 30–34, and 35+), maternal and paternal highest level of education (none, primary, secondary, and higher), paternal and maternal occupation (employed, unemployed, farmer, and others), marital status (married, single, and widowed/divorced), the current area of residence (rural, urban), body mass index (BMI) in kg/m^2^ (normal [18.5–24.9], underweight [<18.5], overweight [25–29.9], and obese [30+]), and paternal age [15–24, 25–29, 30–34, 35+].

Maternal conditions and complications during pregnancy and delivery were number of antenatal care visits (4+, <4), parity (primipara, multipara), HIV status (positive, negative), and referral status (Yes, No). Maternal anemia and malaria during pregnancy, infections, pre-eclampsia/eclampsia, premature rupture of the membranes (PROM), postpartum hemorrhage (PPH), abruption placenta, and placenta previa were all binary (Yes, No). Other information included sex of the child (male, female), birth weight (normal [≥2,500 g], low birth weight (LBW) [<2,500 g]) (35), presentation at birth (cephalic, breech, and transverse), mode of delivery (vaginal, cesarean section [CS]), and Apgar score at 5 min (high [7+], low [<7]).

### 2.4. Data Management and Statistical Analysis

#### 2.4.1. Descriptive Analysis

Data were analyzed using STATA version 15.1 (StataCorp LLC, College Station, Texas, USA) (36). The primary unity of analysis was singleton deliveries for women recorded in the KCMC Medical Birth Registry between 2000 and 2017. We summarized numeric variables using means and standard deviations and categorical variables using frequencies and percentages. The chi-square test compared the proportion of preterm births and perinatal deaths by maternal and paternal background characteristics and maternal conditions and complications during pregnancy and childbirth. Ordinary least-squares linear regression assessed linear trends of proportions of the two outcomes for every year increase. Findings from previous analyses for the predictors of preterm birth (37) and perinatal death (14, 15) informed selection of variables to include in the initial steps of multivariable analysis. The next step was a separate stepwise manual reduction of variables not significantly associated with preterm birth and perinatal death (*p* < 0.05) using the mixed-effects generalized linear models with exchangeable correlation structure. This step was essential given additional variables, such as paternal characteristics, which were significant predictors of perinatal death in the previous analysis using machine learning models (15). Of importance, we tested the effect of including paternal characteristics in this step, which were not significant predictors of any of the two outcomes.

#### 2.4.2. The Joint Model of Two Binary Responses

Joint modeling of preterm birth and perinatal death was achieved using random effects models with an exchangeable correlation structure. Both outcomes were binary, hence used the binomial family and logit link function. We assumed that a set of latent, unobserved random effects of the same mother's two outcomes are correlated. Therefore, we used shared random intercepts to determine the correlation between the same mother's two outcomes, that is, preterm birth and perinatal death. The random intercept captures the unobserved factors specific to each individual, which may influence the responses (26). Let *Y*_*ij*_ denote the *j*th response (*j* = 1, 2) of the *i*th (*i* = 1, 2, …, *n*) subject, with *j* = 1 for preterm birth and *j* = 2 for perinatal death. Also, let *k* (*k* = 1, 2, …, *K*) denote the number of singleton births from mother *i* in the database. A binary response *Y*_*ijk*_ takes the values 1 if an event has occurred and 0 if otherwise. Thus, for the *i*th subject, we have a bivariate binary response vector (*Y*_1*ik*_, *Y*_2*ik*_). We also let *X*_1*i*_ and *X*_2*i*_ represent the vectors of covariates associated with preterm birth and perinatal death, and $\beta_{1}\left(\hat{\beta_{1}}\right)$ and $\beta_{2}\left(\hat{\beta_{2}}\right)$ be their corresponding regression coefficients and estimates in brackets, respectively. Random effects models are used to jointly model two longitudinal outcomes of different nature (26, 27, 29, 38), also referred as multivariate longitudinal models (39). Although the association between the covariates and each outcome (preterm birth and perinatal death) can be examined using separate regression models for each outcome (26), these traditional logistic regression models ignore the correlation between them (26). This study applied the shared parameter random-effects logit model and random-effects multinomial regression models for co-occurrence to determine the joint predictors of preterm birth and perinatal death. The random effects capture the unobserved factors specific to each individual, which may influence the responses (26).

#### 2.4.3. Shared-Parameter Models

The joint model is built by describing the joint density *f*(*y*_1*ik*_, *y*_2*ik*_) of the binary response vectors *Y*_1*ik*_ and *Y*_2*ik*_. Let *b*_*i*_ denote the random effects shared by the two responses of the *i*th individual. We further let *d*_1*jk*_ and *d*_2*jk*_ define the dummy variables, with *d*_1*jk*_ = 1 for *j* = 1 and *d*_2*jk*_ = 1 for *j* = 2. A popular approach is to postulate a so-called shared-parameter model (39), where the joint density for (*Y*_1*ik*_, *Y*_2*i*_) is obtained from


(1) $$\begin{array}{l} f\left(y_{1ik},y_{2ik}\right)=\int f\left(y_{1ik},y_{2ik}|b_{i}\right)f\left(b_{i}\right)db\\ \;=\int f\left(y_{1ik}|b_{i}\right)f\left(y_{2ik}|b_{i}\right)f\left(b_{i}\right)db_{i} \end{array}$$

in which *f*(*b*_*i*_) denotes the random-effects density. The joint response model using logit link for binary responses can be given by (26).


(2) $$\begin{array}{l} \begin{array}{l} logit\left\{E\left(Y_{jik}|b_{i}\right)\right\}=logit\left\{Pr\left(Y_{jik}=1|X_{ji},\beta_{j},b_{i}\right\}\right)\\ \;=d_{1ik}\left(\beta_{1}^{T}X_{1i}+b_{i}\right)+d_{2ik}\left(\beta_{2}^{T}X_{2i}+b_{i}\right) \end{array} \end{array}$$

Alternatively, Equation (2) can be expressed in a vector form as


(3) $$\begin{array}{l} logit\left\{E\left(\begin{array}{c} Y_{1ik}\\ Y_{2ik} \end{array}\right)\right\}=\left(\begin{array}{c} \beta_{1}^{T}X_{1i}+b_{i}\\ \beta_{2}^{T}X_{2i}+b_{i} \end{array}\right) \end{array}$$

where the bivariate responses (*Y*_1*ik*_, *Y*_2*ik*_) of all individuals are stacked into a single response vector (*Y*_*ji*_), where *Y*_*ji*_ = (*Y*_*ji*1_, *Y*_*ji*2_, …, *Y*_*ji*_*k*__*i*__). The random effect *b*_*i*_ is a “shared parameter” inducing correlation between the two binary responses *Y*_1*i*_ and *Y*_2*i*_ through the joint dependence on *b*_*i*_. The conditional independence of *Y*_1*i*_ and *Y*_2*i*_ given *b*_*i*_ may reflect the belief that a common set of underlying characteristics of the individual governs both outcomes (39). The random intercept *b*_*i*_ in 3 shared by both outcomes dictates that correlations between parts of measurements from different outcomes are equal to the product of the correlation between measurements of the two outcomes. In addition, the correlation of deliveries within the mother was accounted for using the exchangeable correlation structure with a robust variance estimator.

#### 2.4.4. Estimation and Inference

The joint responses of *Y*_1*ik*_ and *Y*_2*ik*_ are assumed to be independent given the shared random effects (*b*_*i*_). Assume the *b*_*i*_ are normally distributed with zero mean and variance covariance matrix *D*. Given this assumption, we can write the likelihood function of the joint response model as follows:


(4) $$\begin{array}{l} L(\theta )=\prod_{i=1}^{n}\prod_{j=1}^{2}\int \left\{E(Y_{ji}|X_{ji},\beta_{j},\psi ,b_{i})\right\}dF(b_{i})\\ \;=\prod_{i=1}^{n}\prod_{j=1}^{2}\int \{\prod_{k=1}^{K_{i}}Pr(Y_{jik}=1|X_{jik},\beta_{j},\psi ,b_{i})\}dF(b_{i})\\ \;=\prod_{i=1}^{n}\prod_{j=1}^{2}\int \{\prod_{k=1}^{K_{i}}\frac{e^{b_{i}+\beta_{j}^{\text{T}}X_{jik}}}{1+e^{b_{i}+\beta_{j}^{\text{T}}X_{jik}}}\}dF(b_{i}) \end{array}$$

where θ = (β, ψ) is the vector of all parameters in the conditional distribution and the multivariate normal distribution for *b*_*i*_, *X*_*ji*_ = (*X*_*ji*1_, *X*_*ji*2_, …, *X*_*ji*_*k*__*i*__) corresponds to a vector of covariates associated with preterm birth and perinatal death. *F*(·) is the distribution function of shared random effect *b*_*i*_. β are regression coefficients and ψ contains the variance and covariance parameters for the random effects. The integrals involved in Equation (4) cannot be calculated analytically and numerical approaches are needed (26, 29). Numeric approximations, such as adaptive Gaussian quadrature, are recommended to estimate the model parameters (27, 29, 40, 41). The higher the order of the quadrature, the better the approximation will be of the *N* subjects integrals in the likelihood (40). Once the model has been fitted, inferences for all elements in θ become available using standard likelihood theory (e.g., likelihood ratio tests, Wald tests, score tests) (29).

We used maximum likelihood estimation using adaptive Gaussian quadrature method based on 10 quadrature points to obtain parameter estimates of the joint models (26, 29, 40). This method gives precise parameter estimates at the price of being computationally intensive (40).

#### 2.4.5. A Random-Effects Multinomial Regression Model for Co-occurrence

Two additional multinomial random-effects models were used to assess predictors of preterm birth and perinatal death co-occurrence. These models provided additional information to understand the dependence between the two outcomes conditional on the random effects. The first model was random effects, multinomial regression model, with robust standard errors. Robust standard errors estimation is a commonly applied method of correcting variance–covariance estimates in the presence of clustering (42). As previously explained in section 2.3, we assessed predictors of both outcomes occurring, the occurrence of preterm birth only and perinatal death only, in a single multinomial variable. This model estimated a single random effects variance to account for mother-to-mother variability of the two responses. Let *Y*_*ij*_ denote a nominal response variable for the *i*th subject and *j*th measurement occasion. Given the shared random effects (*b*_*i*_), the probability that a response *Y*_*ij*_ occurs in category *c* for a given level-2 unit (*i*) allowing for any possible set of *C*−1 response categories is written as


(5) $$\begin{array}{l} P_{ijc}=\frac{\exp\left(\eta_{ijc}\right)}{\overset{C}{\underset{c=1}{\sum }}\exp\left(\eta_{ijc}\right)}\;\text{for }c=1,2,\ldots ,C \end{array}$$

where the multinomial logit linear predictor, $\eta_{ijc}=X_{ijc}^{T}\beta_{c}+Z_{ijc}^{T}b_{i}$. The random effects *b*_*i*_ are shared across the *C*−1 binary comparisons in the multinomial logit model. The second model was developed similar to in 5, but allowing for separate but correlated random effects of the multinomial logits. The random effects *b*_*i*_ in the linear predictor, $\eta_{ijc}=X_{ijc}^{T}\beta_{c}+Z_{ijc}^{T}b_{ic}$ are now different for each binary comparison in the multinomial logit. A model with separate random effects estimated covariance parameters for each pair of the multinomial outcomes.

## 3. Results

### 3.1. Preterm Birth and Perinatal Death Proportions by Maternal and Paternal Characteristics

The overall proportions of preterm birth and perinatal death between 2000 and 2017 recorded in the KCMC medical birth registry were 12.8 and 4.3%, respectively, and perinatal mortality rate (PMR) of 42.6 per 1,000 births. The proportions of preterm birth and perinatal death differed significantly (*p* < 0.05) by maternal and paternal background characteristics and obstetric care characteristics (Tables 1, 2). The preterm birth proportion was significantly higher among mothers aged 15–19 (15.7%) and 40+ years (17%), those with no education (16.3%), farmers (16.6%), and rural residents (14.3%). The highest proportions of preterm birth were among younger fathers, that is, 15–24 years (16.2%), with no education (20.5%), and farmers (17.9%). Furthermore, the perinatal death proportions were significantly higher among mothers aged 40+ years (6.4%), with no education (9%), farmers (6.5%), and rural residents (5.6%). Among fathers, perinatal death proportions were high among those aged 30–34 (4.1%) and 35+ years (4.8%), with no education (12.7%), and farmers (7.7%) (Table 1).

**Table 1:** Distribution of preterm birth and perinatal death by maternal and paternal characteristics (*N* = 55,907).

**Characteristics**	**Preterm birth**			**Perinatal death**		
	**Total (%)**	***n* (%)**	***P*-value**	**Total (%)**	***n* (%)**	***P*-value**
**Maternal age in years**			<0.001			<0.001
15–19	3,749 (6.7)	589 (15.7)		44,16 (7.9)	173 (3.9)	
20–24	11,930 (21.4)	1,547 (13.0)		13,648 (24.5)	520 (3.8)	
25–34	25,687 (46.0)	2,973 (11.6)		28,920 (51.8)	1,199 (4.1)	
35–39	6,045 (10.8)	862 (14.3)		6,792 (12.2)	359 (5.3)	
40+	1,609 (2.9)	274 (17.0)		1,851 (3.3)	119 (6.4)	
**Maternal highest education level**			<0.001			<0.001
None	718 (1.3)	117 (16.3)		1,031 (1.8)	93 (9.0)	
Primary	25,669 (46.0)	3,713 (14.5)		29,479 (52.8)	1,500 (5.1)	
Secondary	6,922 (12.4)	914 (13.2)		7,768 (13.9)	244 (3.1)	
Higher	15,727 (28.2)	1,499 (9.5)		17,343 (31.1)	511 (2.9)	
**Maternal occupation**			<0.001			<0.001
Employed	26,226 (47.2)	2,921 (11.1)		29,303 (52.7)	971 (3.3)	
Unemployed	10,445 (18.8)	1,451 (13.9)		11,852 (21.3)	517 (4.4)	
Farmer	9,067 (16.3)	1,501 (16.6)		10,699 (19.3)	700 (6.5)	
Others	3,114 (5.6)	360 (11.6)		3,554 (6.4)	151 (4.2)	
**Marital status**			<0.001			0.08
Married	42,385 (76.0)	5,232 (12.3)		48,037 (86.1)	2,036 (4.2)	
Single	6,569 (11.8)	988 (15.0)		7,477 (13.4)	304 (4.1)	
Widowed/Divorced	89 (0.2)	25 (28.1)		107 (0.2)	9 (8.4)	
**Current area of residence**			<0.001			<0.001
Urban	29,417 (52.8)	3,448 (11.7)		32,915 (59.0)	1,086 (3.3)	
Rural	19,576 (35.1)	2,801 (14.3)		22,673 (40.7)	1,276 (5.6)	
**Body mass index categories (kg/m** ^**2**^ **)**			<0.001			0.64
Normal (18.5–24.9)	18,021 (46.8)	2,029 (11.3)		20,427 (53.0)	696 (3.4)	
Underweight (<18.5)	1,766 (4.6)	232 (13.1)		2,029 (5.3)	68 (3.4)	
Overweight (25–29.9)	9,596 (24.9)	944 (9.8)		10,770 (28.0)	395 (3.7)	
Obese (30+)	4,601 (11.9)	505 (11.0)		5,171 (13.4)	186 (3.6)	
**Paternal age (years)**			<0.001			<0.001
15–24	4,460 (8.0)	721 (16.2)		5,149 (9.3)	189 (3.7)	
25–29	11,979 (21.6)	1,466 (12.2)		13,595 (24.5)	486 (3.6)	
30–34	14,199 (25.6)	1,662 (11.7)		15,995 (28.8)	656 (4.1)	
35+	18,179 (32.8)	2,363 (13.0)		20,582 (37.1)	996 (4.8)	
**Paternal education level**			<0.001			<0.001
None	365 (0.7)	75 (20.5)		529 (1.0)	67 (12.7)	
Primary	21,163 (38.0)	3,154 (14.9)		24,440 (43.9)	1,302 (5.3)	
Secondary	6,083 (10.9)	851 (14.0)		6,776 (12.2)	233 (3.4)	
Higher	21,358 (38.4)	2,152 (10.1)		23,765 (42.7)	741 (3.1)	
**Paternal occupation**			<0.001			<0.001
Employed	41,695 (74.9)	4,964 (11.9)		46,932 (84.3)	1,756 (3.7)	
Unemployed	878 (1.6)	127 (14.5)		1,005 (1.8)	23 (2.3)	
Farmer	5,637 (10.1)	1009 (17.9)		6,671 (12.0)	515 (7.7)	
Others	764 (1.4)	131 (17.1)		915 (1.6)	50 (5.5)	
Total *n* (%)	49,113	6,263 (12.8)		55,736	2,377 (4.3)	

*Variables may not tally to the total frequencies due to missing values in either the exposure or the outcome of interest*.

**Table 2:** Distribution of preterm birth and perinatal death by maternal conditions and complications during pregnancy and delivery (*N* = 55,907).

**Characteristics**	**Preterm birth**			**Perinatal death**		
	**Total (%)**	***n* (%)**	***P*-value**	**Total (%)**	***n* (%)**	***P*-value**
**Number of ANC visits**			<0.001			<0.001
4+	33,291 (60.5)	2,488 (7.5)		37,619 (68.4)	1,111 (3.0)	
<4	15,087 (27.4)	3,581 (23.7)		17,198 (31.3)	1,161 (6.8)	
**Parity**			<0.001			0.23
Multipara	9,456 (16.9)	1,063 (11.2)		10,552 (18.9)	449 (4.3)	
Primipara	39,657 (70.9)	5,200 (13.1)		45,184 (80.8)	1,928 (4.3)	
**Drank alcohol during this pregnancy**			<0.001			0.004
No	35,922 (64.4)	4,778 (13.3)		40,745 (73.0)	1,782 (4.4)	
Yes	13,123 (23.5)	1,474 (11.2)		14,874 (26.7)	568 (3.8)	
**Referred for delivery**			<0.001			<0.001
No	36,498 (67.6)	3,907 (10.7)		40,988 (76.0)	1,169 (2.9)	
Yes	10,910 (20.2)	2,189 (20.1)		12,817 (23.7)	1,092 (8.5)	
**HIV status**			<0.001			0.001
Negative	36,764 (83.7)	4,574 (12.4)		41,568 (94.6)	1,541 (3.7)	
Positive	1,972 (4.5)	304 (15.4)		2,265 (5.2)	114 (5.0)	
**Anemia**			0.54			<0.001
No	48,349 (86.5)	6,160 (12.7)		54,872 (98.1)	2,317 (4.2)	
Yes	764 (1.4)	103 (13.5)		864 (1.5)	60 (6.9)	
**Malaria**			<0.001			0.43
No	42,992 (76.9)	5,600 (13.0)		48,760 (87.2)	2,067 (4.2)	
Yes	6,121 (10.9)	663 (10.8)		6,976 (12.5)	310 (4.4)	
**Any infections condition**			0.07			0.18
No	48,340 (86.5)	6,181 (12.8)		54,869 (98.1)	2,348 (4.3)	
Yes	773 (1.4)	82 (10.6)		867 (1.6)	29 (3.3)	
**Pre-eclampsia/eclampsia**			<0.001			<0.001
No	47,008 (84.1)	5,569 (11.8)		53,389 (95.5)	2,069 (3.9)	
Yes	2,105 (3.8)	694 (33.0)		2,347 (4.2)	308 (13.1)	
**PROM**			<0.001			0.002
No	48,123 (86.1)	6,029 (12.5)		54,635 (97.7)	2,351 (4.3)	
Yes	990 (1.8)	234 (23.6)		1,101 (2.0)	26 (2.4)	
**PPH**			<0.001			<0.001
No	48,760 (87.2)	6,184 (12.7)		55,343 (99.0)	2,304 (4.2)	
Yes	353 (0.6)	79 (22.4)		393 (0.7)	73 (18.6)	
**Abruption placenta**			<0.001			<0.001
No	48,950 (87.6)	6,180 (12.6)		55,552 (99.4)	2,275 (4.1)	
Yes	163 (0.3)	83 (50.9)		184 (0.3)	102 (55.4)	
**Placenta previa**			<0.001			0.08
No	49,002 (87.6)	6,201 (12.7)		55,616 (99.5)	2,368 (4.3)	
Yes	111 (0.2)	62 (55.9)		120 (0.2)	9 (7.5)	
**Sex of the baby**			0.86			0.65
Female	23,664 (42.5)	3,005 (12.7)		26,831 (48.2)	1,128 (4.2)	
Male	25,245 (45.3)	3,219 (12.8)		28,686 (51.5)	1,228 (4.3)	
**Birth weight**			<0.001			<0.001
NBW	43,619 (78.2)	3,313 (7.6)		49,596 (88.9)	1,190 (2.4)	
LBW	5,373 (9.6)	2,889 (53.8)		6,008 (10.8)	1,148 (19.1)	
**Presentation**			<0.001			<0.001
Cephalic	48,160 (86.6)	6,030 (12.5)		54,686 (98.3)	2,183 (4.0)	
Breech	638 (1.1)	173 (27.1)		729 (1.3)	147 (20.2)	
Transverse	75 (0.1)	11 (14.7)		83 (0.1)	17 (20.5)	
**Delivery mode**			<0.001			0.03
Vaginal	32,085 (57.6)	3,744 (11.7)		36,426 (65.4)	1,586 (4.4)	
CS	16,855 (30.3)	2,487 (14.8)		19,116 (34.3)	757 (4.0)	
**Apgar score at 5 min**			<0.001			<0.001
High (7+)	46,015 (83.2)	4,981 (10.8)		52,117 (94.3)	161 (0.3)	
Low (<7)	2,543 (4.6)	1,068 (42.0)		3,006 (5.4)	1,817 (60.4)	
**Induced labor**			<0.001			<0.001
No	37,537 (67.5)	5,088 (13.6)		42,648 (76.6)	1,695 (4.0)	
Yes	11,352 (20.4)	1,135 (10.0)		12,831 (23.1)	667 (5.2)	
Total *n* (%)	49,113	6,263 (12.8)		55,736	2,377 (4.3)	

*Variables may not tally to the total frequencies due to missing values in either the exposure or the outcome of interest*.

### 3.2. Preterm Birth and Perinatal Death Proportions by Maternal Conditions and Complications During Pregnancy and Delivery

The preterm birth proportions were highest among mothers with inadequate (<4) ANC visits (27.4%), those referred for delivery (20.1%), experienced pre-eclampsia/eclampsia (33%), PROM (23.6%), PPH (22.4%), abruption placenta (50.9%), and placenta previa (55.9%), delivered LBW baby (53.8%), experienced breech presentation at birth (27.1%), had <7 5-min Apgar score (42.0%), and experienced perinatal death (47%). Also, the perinatal death proportions are high among mothers with inadequate ANC visits (6.8%), referred for delivery (8.5%), experienced pre-eclampsia/eclampsia (13.1%), PPH (18.6%), delivered LBW baby (19.1%), breech presentation at birth (20.2%), low (<7) 5-min Apgar score (60.4%), and delivered preterm (15.3%). Notably, the highest proportions of perinatal deaths are among those that experienced abruption placenta (55.4%) and with low (<7) 5-min Apgar score (60.4%) (Table 2).

### 3.3. Trends of Preterm Birth and Perinatal Death Between 2000 and 2017

**Figure 2:**
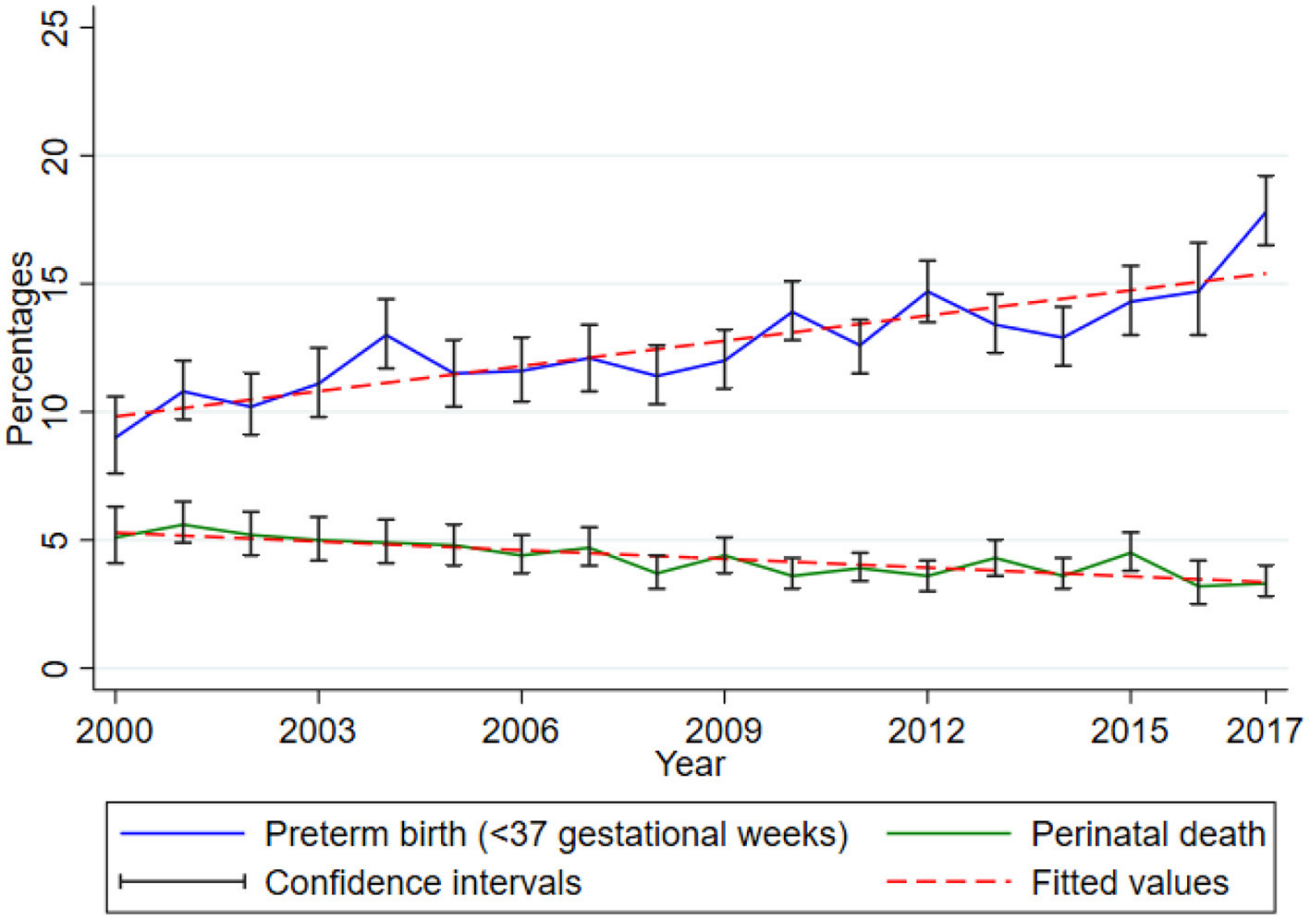
Trends of preterm birth and perinatal death. Data from the Kilimanjaro Christian Medical Center (KCMC) Medical Birth Registry, 2000–2017.

Between 2000 and 2017, there was a rising trend of preterm birth while perinatal death proportions decline slightly in this cohort. The proportion of preterm birth (<37 gestational weeks) increased significantly by 0.33 (95% CI 0.23, 0.43, *p* < 0.001) while that of perinatal death decreased significantly by 0.11 (95% CI 0.08–0.15, *p* < 0.001) for every 1-year increase (Figure 2).

### 3.4. Joint Predictors of Preterm Birth and Perinatal Death

#### 3.4.1. Joint Model With Separate but Correlated Random Effects

Findings of the joint model with separate but correlated random effects are in Table 3. The random-effects variance is observed to be equal for both outcomes (Var = 0.18, 95% CI 0.004, 9.09) and is significantly greater than zero. The covariance parameter capturing dependence between the two outcomes is not statistically significant (Cov = −0.11, 95% CI −0.42, 0.20). Therefore, the two outcomes are independent conditional on accounting for mother to mother variability/heterogeneity.

**Table 3:** Joint predictors of preterm birth and perinatal death with separate but correlated random effects.

**Variables**	**Preterm birth^**†**^**	**Perinatal death4^**‡**^**
	**OR^**¶**^ (95%CI)**	**OR^**¶**^ (95%CI)**
**Maternal age groups**		
15–19	1.24 (1.09, 1.42)***	0.43 (0.30, 0.62)***
20–24	1.16 (1.07, 1.26)***	0.95 (0.77, 1.16)
25–29	1.00	1.00
35–39	1.07 (0.97, 1.19)	1.21 (0.93, 1.57)
40+	1.22 (1.03, 1.46)*	1.30 (0.83, 2.02)
**Maternal education**		
None	1.06 (0.81, 1.39)	-
Primary	1.29 (1.19, 1.40)***	-
Secondary	1.12 (1.00, 1.25)*	-
Higher	1.00	-
Area of residence (Rural)	0.91 (0.84, 0.97)**	-
ANC visits (<4)	2.92 (2.71, 3.15)***	1.26 (1.05, 1.51)*
Referred for delivery (Yes)	1.32 (1.21, 1.43)***	1.37 (1.13, 1.66)**
Parity (Primipara)	-	0.69 (0.53, 0.89)**
Pre-eclampsia/eclampsia (Yes)	1.79 (1.56, 2.05)***	1.05 (0.79, 1.40)
PROM (Yes)	1.92 (1.59, 2.33)***	0.35 (0.17, 0.72)**
PPH (Yes)	-	3.46 (2.02, 5.93)***
Abruption placenta (Yes)	1.73 (1.03, 2.92)*	2.43 (1.35, 4.40)**
Placenta previa (Yes)	4.71 (2.66, 8.35)***	0.21 (0.06, 0.79)*
Sex (Male)	1.12 (1.04, 1.19)**	0.83 (0.71, 0.98)*
LBW (Yes)	10.36 (9.11, 11.77)***	1.32 (0.97, 1.78)
**Presentation at birth**		
Cephalic	1.00	1.00
Breech	1.54 (1.19, 1.99)**	4.01 (1.96, 8.19)***
Transverse	0.83 (0.31, 2.26)	20.46 (2.79, 149.75)**
Delivery mode (CS)	1.06 (0.98, 1.14)	0.58 (0.46, 0.72)***
Five minutes Apgar score (<7)^¶¶^	2.29 (2.04, 2.57)***	496.61 (240.07, 1027.28)***
Induced labor (Yes)	0.82 (0.75, 0.89)***	1.43 (1.18, 1.73)***
Year	1.04 (1.03, 1.05)***	0.97 (0.95, 0.99)*
Variance of the random effects	0.18 (0.004, 9.09)	0.18 (0.004, 9.09)
Covariance	-0.11 (-0.42, 0.20)	

*^†^N = 45,320;^‡^N = 45,378*.

*Maternal education level and area of residence were not significant predictors of perinatal death. At the same time, parity and PPH were not significant predictors of preterm birth, hence not included in the final model*.

*^¶^OR: Odds ratios adjusted for maternal age, education level, area of residence, number of ANC visits, referral status, parity, pre-eclampsia/eclampsia, PROM, PPH, abruption placenta, placenta previa, sex of the child, LBW, presentation at birth, delivery mode, 5-min Apgar score, labor induction, and year of birth. Parity and PPH were not included in preterm birth model while education level not included in perinatal death prediction*.

*^¶¶^Odds ratio not estimable due to very small number of perinatal deaths among mothers who delivered children with 5-min Apgar score of 7 and above. Too wide confidence intervals demonstrate low precision of parameter estimates, except for preterm birth only*.

*p < 0.05, **p < 0.01, ***p < 0.001.

Conditional on the random-effects, inadequate (<4) ANC visits (OR = 2.92, 95% CI 2.71, 3.15 and OR = 1.26, 95% CI 1.05, 1.51), being referred for delivery (OR = 1.32, 95% CI 1.21, 1.43, and OR = 1.37, 95% CI 1.13, 1.66), abruption placenta (OR = 1.73, 95% CI 1.03, 2.92 and OR = 2.43, 95% CI 1.35, 4.40), and breech presentation (OR = 1.54, 95% CI 1.19, 1.99 and OR = 4.01, 95% CI 1.96, 8.19) increased the odds of both preterm birth and perinatal death, respectively. For every 1-year increase, we expect to see the odds of preterm birth increasing significantly by 4% (OR = 1.04, 95% CI 1.03, 1.05) and 3% decrease in perinatal death (OR = 0.97, 95% CI 0.95, 0.99).

Also, conditional on the random effects, adolescent mothers (15–19 years) were significantly more likely to deliver preterm (OR = 1.24, 95% CI 1.09, 1.42) but had lower odds of experiencing perinatal death (OR = 0.43, 95% CI 0.30, 0.62). Similar results were among mothers aged 20–24 years, though this association was not statistically significant. Likewise, higher odds of preterm birth were among mothers who experienced PROM (OR = 1.92, 95% CI 1.59, 2.33), experienced placenta previa (OR = 4.71, 95% CI 2.66, 8.35), and among male children (OR = 1.12, 95% CI 1.04, 1.19). On the contrary, experiencing PROM (OR = 0.35, 95% CI 0.17, 0.72) and placenta previa (OR = 0.21, 95% CI 0.06, 0.79), and male children (OR = 0.83, 95% CI 0.71, 0.98) were less likely to experience perinatal death. Induction of labor was protective of preterm birth of preterm birth (OR = 0.82, 95% CI 0.75, 0.89) but increased the odds of perinatal death (OR = 1.43, 95% CI 1.18, 1.73).

#### 3.4.2. Predictors of Co-occurrence of Preterm Birth and Perinatal Death Using Random Effect Multinomial Regression Model

Findings from the random-effect multinomial regression model are presented in Table 4. This model's random-effects variance is not significantly from zero (Var = 0.04, 95% CI 0.00, 99.43). The observed results are not surprising. The reason is that we generated a multinomial variable from preterm birth and perinatal death, allowing for modeling the dependence between the two outcomes directly other than through separate and correlated random effects. Significantly higher odds of co-occurrence of preterm birth and perinatal death were among mothers with inadequate (<4) ANC visits (OR = 3.46, 95% CI 2.77, 4.32), experienced pre-eclampsia/eclampsia (OR = 1.38, 95% CI 1.01, 1.89), PPH (OR = 2.24, 95% CI 1.05, 4.78), and abruption placenta (OR = 3.98, 95% CI 2.02, 7.82), delivered LBW baby (OR = 12.81, 95% CI 9.84, 16.67), and had a breech presentation (OR = 3.79, 95% CI 2.03, 7.08). Adolescent mothers (15–19 years) (OR = 0.46, 95% CI 0.29, 0.73), with no education (OR = 0.41, 95% CI 0.20, 0.84), primipara (OR = 0.63, 95% CI 0.48, 0.84), and delivered through CS (OR = 0.50, 95% CI 0.40, 0.64) had lower odds of co-occurrence of preterm birth and perinatal death.

**Table 4:** Predictors of independent and co-occurrence of preterm birth and perinatal death using random effect multinomial regression model (*N* = 51,493).

**Variable**	**Co-occurrence^**†**^**	**Perinatal death only**	**Preterm birth only**
	**OR^**‡**^ (95%CI)**	**OR^**‡**^ (95%CI)**	**OR^**‡**^ (95%CI)**
**Maternal age groups**			
15–19	0.46 (0.29, 0.73)**	0.56 (0.40, 0.79)***	1.24 (1.09, 1.42)***
20–24	1.10 (0.85, 1.43)	1.00 (0.81, 1.24)	1.16 (1.07, 1.26)***
25–29	1.00	1.00	1.00
35–39	1.35 (0.99, 1.84)	1.20 (0.92, 1.56)	1.07 (0.96, 1.19)
40+	1.60 (0.95, 2.68)	1.02 (0.65, 1.61)	1.20 (1.00, 1.43)*
**Maternal education**			
None	0.41 (0.20, 0.84)*	0.67 (0.39, 1.16)	0.68 (0.51, 0.91)**
Primary	0.93 (0.72, 1.21)	1.09 (0.88, 1.35)	1.27 (1.16, 1.38)***
Secondary	0.77 (0.53, 1.14)	1.22 (0.90, 1.67)	1.14 (1.02, 1.27)*
Higher	1.00	1.00	1.00
Area of residence (Rural)	0.86 (0.69, 1.07)	0.93 (0.77, 1.11)	0.89 (0.82, 0.96)**
ANC visits (<4)	3.46 (2.77, 4.32)***	1.11 (0.92, 1.33)	2.79 (2.58, 3.00)***
Referred for delivery (Yes)	1.27 (1.00, 1.61)	1.23 (1.00, 1.52)*	1.28 (1.18, 1.40)***
Parity (Primipara)	0.63 (0.48, 0.84)**	0.75 (0.60, 0.95)*	0.99 (0.91, 1.09)
Pre-eclampsia/eclampsia (Yes)	1.38 (1.01, 1.89)*	1.24 (0.90, 1.72)	1.76 (1.53, 2.02)***
PROM (Yes)	0.85 (0.34, 2.13)	0.39 (0.18, 0.88)*	2.01 (1.66, 2.43)***
PPH (Yes)	2.24 (1.05, 4.78)*	3.16 (1.87, 5.33)***	0.96 (0.65, 1.42)
Abruption placenta (Yes)	3.98 (2.02, 7.82)***	2.21 (1.15, 4.24)*	1.75 (0.94, 3.27)
Placenta previa (Yes)	0.60 (0.12, 2.95)	1.39 (0.27, 7.17)	5.32 (3.09, 9.16)***
Sex (Male)	0.88 (0.71, 1.08)	0.95 (0.80, 1.13)	1.12 (1.04, 1.19)**
LBW (Yes)	12.81 (9.84, 16.67)***	1.00 (0.79, 1.26)	9.57 (8.48, 10.80)***
**Presentation at birth**			
Cephalic	1.00	1.00	1.00
Breech	3.79 (2.03, 7.08)***	2.58 (1.39, 4.77)**	1.25 (0.93, 1.68)
Transverse	10.01 (0.49, 204.86)	25.63 (4.76, 137.90)***	1.07 (0.39, 2.90)
Delivery mode (CS)	0.50 (0.40, 0.64)***	0.72 (0.59, 0.87)***	1.11 (1.03, 1.19)**
Five minutes Apgar score (<7)^¶^	466.88 (294.54, 740.05)***	351.03 (261.03, 472.04)***	1.07 (0.81, 1.40)
Induced labor (Yes)	1.23 (0.96, 1.58)	1.34 (1.10, 1.64)**	0.81 (0.74, 0.89)***
Year at birth	1.02 (1.00, 1.05)	0.98 (0.96, 1.00)	1.04 (1.04, 1.05)***
Variance of the random effect	0.04 (0.00, 99.43)		

*^†^Co-occurrence means the occurrence of both preterm birth and perinatal death*.

*^‡^OR: Odds ratios adjusted for maternal age, education level, area of residence, number of ANC visits, referral status, parity, pre-eclampsia/eclampsia, PROM, PPH, abruption placenta, placenta previa, sex of the child, LBW, presentation at birth, delivery mode, 5-min Apgar score, labor induction, and year of birth*.

*^¶^Odds ratio not estimable due to very small number of perinatal deaths among mothers who delivered children with 5-min Apgar score of seven and above. Too wide confidence intervals demonstrates low precision of parameter estimates, except for preterm birth only (last column)*.

*p < 0.05, **p < 0.01, ***p < 0.001.

#### 3.4.3. Predictors of Co-occurrence of Preterm Birth and Perinatal Death Using a Multinomial Regression Model With Separate but Correlated Random Effects

Results of the joint model presented in Table 4 have a single variance component for the three multinomial outcomes. Table 5 contains findings of a multinomial regression model with separate but correlated random effects. The variance components indicate high variability for the co-occurrence of both outcomes (Var = 1.23, 95% CI 0.20, 7.60) than the outcomes occurring independently (Var = 0.70, 95% CI 0.04, 11.5 and Var = 0.50, 95% CI 0.28, 0.90, for perinatal death and preterm birth, respectively). The covariance between a pair of these outcomes gives no evidence of dependence, conditional on accounting for mother to mother variability. Furthermore, we also observed relatively larger standard errors (especially for the co-occurrence and perinatal death only) for this model (standard errors not shown) than the model with a single variance component. The confidence intervals for the predictors of co-occurrence and perinatal death in Table 4 are relatively narrow compared to those in Table 5. Results show no correlation between random effects in this analysis, hence used BIC for model comparison (43). Therefore, model comparison using BIC agreed with the results mentioned above. Specifically, the model corresponding to results presented in Table 4 had a BIC of 33,798.52, which is smaller than 33,828.32 for the more complex model corresponding to Table 5. Hence, the best model is the random effect multinomial regression model than the one with separate but correlated random effects (more complex, i.e., has additional parameters). However, the BIC values reported should be interpreted with caution because the conditional AIC is the correct information criteria for clustered data modeled using the random effects approach (43–45). STATA, the software of choice used in the current paper, does not currently have options for these post-estimation performance measures.

**Table 5:** Predictors of independent and co-occurrence of preterm birth and perinatal death using multinomial regression model with separate but correlated random effects (*N* = 51,493).

**Variable**	**Co-occurrence^**†**^**	**Perinatal death only**	**Preterm birth only**
	**OR^**‡**^ (95%CI)**	**OR^**‡**^ (95%CI)**	**OR^**‡**^ (95%CI)**
**Maternal age groups**			
15–19	0.40 (0.23, 0.72)**	0.53 (0.35, 0.80)**	1.24 (1.09, 1.42)***
20–24	1.11 (0.82, 1.50)	1.00 (0.79, 1.26)	1.16 (1.07, 1.26)***
25–29	1.00	1.00	1.00
35–39	1.42 (0.99, 2.05)	1.23 (0.91, 1.65)	1.07 (0.96, 1.19)
40+	1.75 (0.94, 3.26)	1.04 (0.63, 1.71)	1.19 (1.00, 1.43)
**Maternal education**			
None	0.36 (0.16, 0.83)*	0.64 (0.34, 1.19)	0.68 (0.51, 0.91)**
Primary	0.91 (0.67, 1.23)	1.09 (0.86, 1.38)	1.27 (1.16, 1.38)***
Secondary	0.74 (0.47, 1.16)	1.23 (0.88, 1.72)	1.14 (1.02, 1.27)*
Higher	1.00	1.00	1.00
Area of residence (Rural)	0.85 (0.65, 1.09)	0.92 (0.75, 1.12)	0.89 (0.82, 0.96)**
ANC visits (<4)	3.97 (2.79, 5.66)***	1.11 (0.88, 1.40)	2.79 (2.58, 3.00)***
Referred for delivery (Yes)	1.30 (0.98, 1.71)	1.26 (0.99, 1.59)	1.28 (1.18, 1.40)***
Parity (Primipara)	0.60 (0.43, 0.85)**	0.72 (0.54, 0.97)*	0.99 (0.91, 1.09)
Pre-eclampsia/eclampsia (Yes)	1.44 (1.00, 2.06)*	1.26 (0.89, 1.79)	1.76 (1.53, 2.02)***
PROM (Yes)	0.87 (0.29, 2.64)	0.36 (0.14, 0.90)*	2.01 (1.66, 2.43)***
PPH (Yes)	2.44 (1.02, 5.87)*	3.40 (1.92, 6.00)***	0.96 (0.65, 1.41)
Abruption placenta (Yes)	4.80 (2.16, 10.67)***	2.46 (1.11, 5.43)*	1.77 (0.93, 3.34)
Placenta previa (Yes)	0.45 (0.07, 2.96)	1.38 (0.24, 7.80)	5.36 (3.11, 9.25)***
Sex (Male)	0.86 (0.68, 1.10)	0.95 (0.79, 1.15)	1.12 (1.04, 1.20)**
LBW (Yes)	17.00 (9.51, 30.36)***	1.01 (0.60, 1.70)	9.60 (8.51, 10.83)***
**Presentation at birth**			
Cephalic			
Breech	4.60 (2.01, 10.53)***	2.86 (1.30, 6.28)**	1.25 (0.93, 1.69)
Transverse	12.14 (0.50, 296.70)	28.07 (5.64, 139.69)***	1.06 (0.39, 2.86)
Delivery mode (CS)	0.45 (0.34, 0.60)***	0.69 (0.52, 0.90)**	1.11 (1.03, 1.19)**
Five minutes Apgar score (<7)^¶^	750.47 (228.10, 2469.12)***	457.69 (184.43, 1135.84)***	1.05 (0.80, 1.38)
Induced labor (Yes)	1.26 (0.95, 1.66)	1.38 (1.10, 1.72)**	0.81 (0.74, 0.89)***
Year at birth	1.02 (0.99, 1.06)	0.98 (0.95, 1.00)	1.04 (1.04, 1.05)***
Variance of the random effects	1.23 (0.20, 7.60)	0.70 (0.04, 11.5)	0.50 (0.28, 0.90)
Covariances			
Cov(1,2)	0.54 (−0.78, 2.86)		
Cov(1,3)	0.12 (−0.51, 0.74)		
Cov(2,3)	0.20 (−0.34, 0.73)		

*^†^Co-occurrence means the occurrence of both preterm birth and perinatal death*.

*^‡^OR: Odds ratios adjusted for maternal age, education level, area of residence, number of ANC visits, referral status, parity, pre-eclampsia/eclampsia, PROM, PPH, abruption placenta, placenta previa, sex of the child, LBW, presentation at birth, delivery mode, 5-min Apgar score, labor induction, and year of birth*.

*^¶^Odds ratio not estimable due to very small number of perinatal deaths among mothers who delivered children with 5-min Apgar score of 7 and above. Too wide confidence intervals demonstrates low precision of parameter estimates, except for preterm birth only (last column)*.

*p < 0.05, **p < 0.01, ***p < 0.001.

Similar to the descriptions of results in section 3.4.2, conditional on separate random effects for each outcome in the multinomial logits, significantly higher odds of co-occurrence of preterm birth and perinatal death were among mothers with inadequate (<4) ANC visits (OR = 3.97, 95% CI 2.79, 5.66), experienced pre-eclampsia/eclampsia (OR = 1.44, 95% CI 1.00, 2.06), PPH (OR = 2.44, 95% CI 1.02, 5.87), and abruption placenta (OR = 4.80, 95% CI 2.16, 10.67), delivered LBW baby (OR = 17.00, 95% CI 9.51, 30.36), and had a breech presentation (OR = 4.60, 95% CI 2.01, 10.53). Adolescent mothers (15–19 years) (OR = 0.40, 95% CI 0.23, 0.72), with no education (OR = 0.36, 95% CI 0.16, 0.83), primipara (OR = 0.60, 95% CI 0.43, 0.85), and delivered through CS (OR = 0.45, 95% CI 0.34, 0.60) had lower odds of co-occurrence of preterm birth and perinatal death.

## 4. Discussion

The study aimed to determine the joint predictors of preterm birth and perinatal death based on the birth cohort data from the KCMC zonal referral hospital in Northern Tanzania between 2000 and 2017. Conditional on the random effects, higher odds of both preterm birth and perinatal death were among mothers with inadequate (<4) ANC visits, referred for delivery, experienced abruption placenta, and breech presentation. Mothers with inadequate ANC visits, who experienced pre-eclampsia/eclampsia, PPH, and abruption placenta, delivered LBW, and experienced breech presentation had a higher likelihood of co-occurring both preterm birth and perinatal death. Lower odds of co-occurrence were among adolescent mothers (15–19), with no education, primipara, and those delivered through CS.

There is notable progress in reducing neonatal mortality rates in Tanzania (7, 46, 47). However, by 2015, there was slower progress in maternal and newborn survival in Tanzania (46). Despite interventions implemented prior the MDG era (48), early neonatal mortality rates have been on the rise (7, 14, 48). The KCMC Medical Birth registry data demonstrates a slowly declining trend of perinatal deaths (which includes early neonatal deaths). Still, these trends should be interpreted with caution given the potential under-reporting of perinatal deaths events in this registry (14, 49). Appropriate interventions to reduce the rising preterm birth rates are necessary (37) given its known contribution to perinatal and neonatal deaths (1, 48). The UN Inter-agency Group for Child Mortality Estimation indicated that “the focus should be on maintaining high coverage of quality antenatal care, skilled care at birth, postnatal care for mother and baby, and care of small and sick newborns to address the main causes of neonatal mortality globally” (1).

Inadequate ANC visits increased the risk of both preterm birth and perinatal death. Previous studies on independent predictors of these outcomes support this finding (14, 16, 17, 37, 50, 51). A separate analysis including an interaction term between the number of antenatal care visits and maternal age groups in the co-occurrence model (results not shown) was statistically significant for preterm birth only for mothers aged 15–19 and 40+ compared to 25–29 years. Nevertheless, the direction of association for all interaction terms was the same, suggesting that ANC attendance among pregnant women in Tanzania may not depend on maternal age. According to WHO, “within the continuum of reproductive health care, ANC provides a platform for important health-care functions, including health promotion, screening and diagnosis, and disease prevention” (52). Tanzania's local and national efforts should promote good healthcare-seeking behaviors during pregnancy and improved coverage and quality of antenatal care services at all levels of care (53, 54) regardless of maternal age. It is also essential to improve intrapartum and postnatal care quality, particularly for women who experienced pregnancy and delivery-related complications (1, 4, 55, 56).

Women referred for delivery had higher odds of preterm birth and perinatal death. Pregnant women referred for delivery are more likely to experience delivery-related complications, where adolescent mothers have elevated risk (57). In this study, adolescent mothers (15–19 years), primipara, and those with no education were less likely to experience co-occurring preterm birth and perinatal death. The joint random effects model (conditional on the mother-to-mother variability) revealed that those aged 15–19 and 20–24 years were more likely to deliver preterm but had lower odds of perinatal death. However, the protective effect of 20–24 years of age on the risk of perinatal death was not statistically significant. CS delivery lowered the odds of co-occurrence, which may reflect timely care of these high-risk pregnancies to save both the mother and child's life.

Conditional on the random effects, significantly higher odds of preterm birth and perinatal death, and co-occurrence were among mothers who experienced abruption placenta and breech presentation. Additionally, pre-eclampsia/eclampsia, PPH, and LBW increased the likelihood of co-occurrence. On top of these complications being among the common risk factors of preterm birth (16, 22, 37, 58) and perinatal death (14, 15, 19, 21, 59), they also increase the risk of newborns transfer to intensive care units (60). Given their history, women at risk of these adverse pregnancy events should be given due public health and clinical attention and care during antenatal, intrapartum, and postnatal periods. Although we did not assess health system performance regarding pregnancy and childcare, efforts are needed to strengthen health facilities providing delivery services in Tanzania for improved pregnancy outcomes (1, 6, 61).

The study had several strengths compared to previous studies. First, this is the first study in Tanzania and potentially in SSA to assess the joint predictors of preterm birth and perinatal death, to the best of our knowledge. The vast majority of previous studies focused on determining the independent predictors of preterm birth and perinatal death or the determinant of each other. Second, joint modeling using random effects approach accounted for the relationship between the two outcomes for improved precision of parameter estimates. Nevertheless, conditional on the random effects, we observed no statistically significant covariance between preterm birth and perinatal death. In other words, the two outcomes are independent conditional on accounting for mother-to-mother variability.

As we explained elsewhere (14, 15, 37), the study has several limitations. Data for this study come from a medical birth registry at the KCMC zonal referral hospital in northern Tanzania, affecting the generalization of findings. However, less than a quarter (23.8%) of all recorded deliveries were referrals. Hence the study findings may reflect prenatal and intrapartum care practices and adverse events among deliveries from women in the hospital's catchment area, similar settings in Tanzania and SSA. Also, the KCMC medical birth registry cohort only captures perinatal deaths occurring in the health facility (KCMC hospital), which may underestimate the reported perinatal death proportions/rates (15). In addition, gestational age was analyzed as a binary variable, ignoring other preterm birth categories (37), which remains an area for future applications in joint modeling of categorical data.

Regular chi-square test was used in descriptive statistics but may be inappropriate where there are repeated measures (62). However, this is precisely the reason to consider using random-effects models for the analysis of repeated measures. Furthermore, BIC was used for model comparison. Although BIC criteria can be used when random effects are uncorrelated (43), as found in this study, the BIC values are correct only when the underlying variance–covariance structure is well-specified. Other information criteria such as the conditional AIC can be used (43–45) but are not currently available in STATA software. Previous analyses related to this work accounted for missing data (14, 37). However, for the joint modeling analysis, despite imputing the missing data, analysis of the imputed data could not be achieved due to model complexity (i.e., having additional parameters to estimate) and the machine's computational power.

## 5. Conclusion

The joint predictors of higher risk of preterm birth and perinatal death were inadequate (<4) ANC visits, referred for delivery, and complications during pregnancy and childbirth, specifically pre-eclampsia/eclampsia, PPH, LBW, abruption placenta, and breech presentation. Younger maternal age (15–24 years), PROM, placenta previa, and male children have higher odds of preterm birth but a lessened likelihood of perinatal death. ANC is a critical entry point for delivering the recommended interventions to pregnant women (52), especially those at high risk of experiencing adverse pregnancy outcomes. Improved management of complications during pregnancy and childbirth and the postnatal period may eventually lead to a substantial reduction of adverse perinatal outcomes and improving maternal and child health.

## Data Availability Statement

The data analyzed in this study is subject to the following licenses/restrictions: The data contains potentially identifying and sensitive patient information. This has also been stipulated by the Local Institutional Review Board of KCMC hospital and the National Ethics Committee in Norway when establishing this birth registry. Permission to use the data in this study was made through the Kilimanjaro Christian Medical University College Research and Ethics Review Committee, and received an approval number 2424. The authors do not have the legal right to share the data publicly. Requests to access these datasets should be directed to the Executive Director of the KCMC hospital, kcmcadmin@kcmc.ac.tz.

## Ethics Statement

As we previously described (14, 15, 37), this study was approved by the Kilimanjaro Christian Medical University College Research Ethics and Review Committee (KCMU-CRERC) with approval number 2424. For practical reasons, since the interview was administered just after the woman had given birth, consent was given orally. The midwife-nurse gave every woman oral information about the birth registry, the data needed to be collected from them, and the use of the data for research purposes. Women were also informed about the intention to gather new knowledge, which will, in turn, benefit mothers, and children in the future. Participation was voluntary and had no implications on the care women would receive. Following consent, mothers were free to refuse to reply to single questions. For privacy and confidentiality, unique identification numbers were used to both identity and then link mothers with child records. There was no any person-identifiable information in any electronic database, and instead, unique identification numbers were used. Necessary measures were taken by midwives to ensure privacy during the interview process.

## Author Contributions

IM, MM, JO, and HM contributed to the acquisition, analysis, or interpretation of the data. IM analyzed the data, drafted the manuscript, and had primary responsibility for the final content. MM, JO, and HM critically reviewed the manuscript. All authors made a substantial contribution to this study and have read and approved the final version to be published.

## Funding

This work was funded by GSK Africa Non-Communicable Disease Open Lab through the DELTAS Africa Sub-Saharan African Consortium for Advanced Biostatistics (SSACAB) Grant No. 107754/Z/15/Z-training programme. The funders had no role in study design, data collection and analysis, decision to publish, or preparation of the manuscript. The views expressed in this publication are those of the author(s) and not necessarily those of GSK.

## Conflict of Interest

The authors declare that the research was conducted in the absence of any commercial or financial relationships that could be construed as a potential conflict of interest.

## Publisher's Note

All claims expressed in this article are solely those of the authors and do not necessarily represent those of their affiliated organizations, or those of the publisher, the editors and the reviewers. Any product that may be evaluated in this article, or claim that may be made by its manufacturer, is not guaranteed or endorsed by the publisher.
